# Severe Undervirilisation in a 46,XY Case Due to a Novel Mutation in HSD17B3 Gene

**DOI:** 10.4274/jcrpe.2069

**Published:** 2015-08-31

**Authors:** Ayfer Alikaşifoğlu, Doğuş Vurallı, Olaf Hiort, Nazlı Gönç, Alev Özön, Nurgün Kandemir

**Affiliations:** 1 Hacettepe University Faculty of Medicine, Department of Pediatrics, Division of Pediatric Endocrinology, Ankara, Turkey; 2 University of Lübeck Faculty of Medicine, Department of Pediatrics, Division of Pediatric Endocrinology and Diabetes, Lübeck, Germany

**Keywords:** 17 beta-hydroxysteroid dehydrogenase type 3, 46, XY disorders of sex development, delta-4-androstenedione

## Abstract

17-β-hydroxysteroid dehydrogenase type 3 (17β-HSD3) is an important enzyme involved in the final steps of androgen synthesis and is required for the development of normal male external genitalia. 46,XY individuals with deficiency of this enzyme present a wide clinical spectrum from a female appearance of the external genitalia through ambiguous genitalia to a predominantly male genitalia with micropenis or hypospadias. This paper reports a one-year-old 46,XY patient with 17β-HSD3 deficiency who presented with female external genitalia and bilaterally palpable gonads in the inguinal region. The low T/Δ4 ratio after human chorionic gonadotropin (hCG) stimulation suggested 17β-HSD3 deficiency. A homozygous mutation, c.761_762delAG, was determined at the intron 9/exon 10 splice site of the HSD17B3 gene. To the best of our knowledge, this mutation has not been reported thus far, but its localization and type would imply a complete disruption of the 17β-HSD3 which may explain the phenotype of our patient.

## INTRODUCTION

17-b-hydroxysteroid dehydrogenase type 3 (17b-HSD3) deficiency (OMIM ♯264300), also previously described as 17-ketosteroid reductase deficiency, is a rare autosomal recessive form of a 46,XY disorder of sex development (DSD) and is the most common testosterone biosynthesis defect (1). The 17b-HSD3 enzyme is found mainly in the testes and is involved in the conversion of Δ4-androstenedione, which is a weak androgen, to testosterone, which is biologically more active. The 17b-HSD3 enzyme family includes at least 14 isoenzymes identified thus far and these isoenzymes contribute to reproductive organ development by playing a role in the final steps of androgen and estrogen syntheses (2).

The actual incidence of 17b-HSD3 deficiency is unknown; however, previous studied have reported that the estimated incidence is 1/147 000 in newborns and that the calculated heterozygote frequency is 1/135 (3). However, higher incidence rates have been observed in places where consanguineous marriage is common, such as Middle Eastern countries (4).

46,XX individuals with a deficiency of this enzyme are asymptomatic and difficult to diagnose since they have normal female genitalia and normal gender roles as well as uninhibited fertility (4,5). On the other hand, 46,XY individuals may present a wide clinical spectrum from completely female appearing external genitalia (Sinnecker type 5) to slightly androgenized (Sinnecker type 4), frankly ambiguous genitalia (Sinnecker type 3) and to predominantly male genitalia with micropenis or hypospadias (Sinnecker type 2) (3,6,7,8,9). Since the clinical findings of 17b-HSD3 deficiency are similar to other 46,XY DSD forms, it may be difficult at times to establish the actual diagnosis and some of the 17b-HSD3 deficiency patients may be inadvertently diagnosed with androgen resistance (androgen insensitivity syndrome) or 5-α-reductase 2 deficiency. 17b-HSD3 deficiency is diagnosed via hormonal evaluation and the diagnosis is confirmed by molecular genetic testing.

Herein, we report the case of a child who presented with bilateral palpable gonads in the inguinal region during infancy and female appearing external genitalia. A 46,XY karyotype was found and, subsequently, the child was diagnosed with 17b-HSD3 deficiency after detecting a lower T/Δ4 ratio in the stimulation test with human chorionic gonadotropin (hCG) and confirmed by molecular genetic analysis of the HSD17B3 gene. This report is presented since 17b-HSD3 deficiency is a rare form of 46,XY DSD and the mutation identified in our case has not been reported so far.

## CASE REPORT

A one-year-old girl was referred with the complaint of swelling in the right inguinal area. There was consanguinity in the family and the parents were first cousins. Physical examination revealed bilateral palpable gonads in the inguinal region. She had a female appearing genital status. A slight clitoral enlargement to 1.5 cm was observed although the vaginal and urethral orifices were separate. On ultrasonography, no Mullerian structures could be seen and the gonads were in the inguinal canal. The karyotype was determined as 46,XY. A hCG stimulation test was performed and following injection of 1500 U/m2 hCG for 3 days, serum androgen concentrations were measured (Table 1). The test results showed that there was impairment in testosterone biosynthesis. Testosterone/dihydrotestosterone ratio was 3.6, i.e. normal value. Testosterone/androstenedione ratio was found to be 0.107 (N>0.8), suggesting 17b-HSD3 deficiency.

Genetic analysis was made in order to confirm the diagnosis and molecular analysis of the HSD17B3 gene showed a homozygous mutation c.761_762delAG corresponding to p.E254VfsX10 in the patient; both parents were heterozygous. This deleterious mutation has likely caused a 17b-HSD3 deficiency in our patient, although this is a new mutation that has not been identified before, to the best of our knowledge. The parents did not accept sex reassignment into male and bilateral gonadectomy was performed. The histopathology of the gonads were consistent with testis and spermatic cord and no malignancy was seen.

## DISCUSSION

The most frequent presentation of 17b-HSD3 deficiency is a 46,XY case with female appearing external genitalia, labial fusion and a blind-ending vagina, with or without clitoromegaly (3). Most cases are not diagnosed at birth since they have female appearing external genitalia and are raised as female and the diagnosis of such cases is delayed until adolescence (9,10,11,12). In pubertal years, these individuals who have been raised as female and have not undergone gonadectomy are only diagnosed when they present with primary amenorrhea or virilization of various degrees such as increased body hair growth, thickened vocal cords, male type of body development and an enlarged clitoris. Sometimes, as is in the current case, individuals present with inguinal hernia and palpable testes in the inguinal canal or in the labiosacrotal folds and are diagnosed during childhood (3,9,11). Less often, individuals with micropenis or hypospadias are considered to be male at birth and raised accordingly (4). The extent of virilizativon of the individuals varies by the partial residual activity of the 17b-HSD3 isoenzyme in the testes and the conversion of androstenedione to testosterone by other isoenzymes found in the extratesticular tissues, such as the 17b-HSD5 isoenzyme (9,11,13,14,15). Virilization occurs also in puberty because of increased Δ4-androstenedione due to gonadotropin surge and increased conversion of androstenedione to testosterone in the extratesticular tissues (12,14). Patients may also suffer pubertal gynecomastia resulting from the conversion of androstenedione to estrogen through the activities of aromatase and other 17b-HSD isoenzymes (16). As in the current case, urethral and vaginal openings are separated in most of the patients with female external genitalia; however, only a short urogenital sinus has been reported in some individuals (11,17,18).

The clinical findings of 17b-HSD3 deficiency are similar to androgen resistance or 5α reductase 2 deficiency and it is clinically difficult to differentiate between these conditions. 17b-HSD3 deficiency is diagnosed via hormonal evaluation and the diagnosis should be confirmed by molecular genetic testing. Typical hormonal finding of 17b-HSD3 deficiency include an increased Δ4 androstenedione and reduced testosterone concentration. Patients can be diagnosed via basal hormone levels in adulthood and in mini puberty during infancy (in infants aged below six months); however, the diagnosis may be missed unless hCG stimulation test is performed outside of these age periods. Our patient had a T/Δ4 ratio <0.8 after hCG stimulation, which strongly suggests a 17b-HSD3 deficiency. However, it should not be forgotten that this ratio may be low in other conditions related to testosterone synthesis such as dysgenetic testes (11,19). Displaying the absence of Mullerian structures and the presence of Wolffian structures using imaging methods is helpful in the diagnosis but remains insufficient since these can be seen in 5α reductase 2 deficiency and androgen receptor mutations as well as 17b-HSD3 deficiency. Histological examination of the specimens from the gonad shows normal testicular structures and thereby, other causes involved in the etiology of 46,XY DSD, such as testicular dysgenesis, are excluded.

The definitive diagnosis of 17b-HSD3 deficiency is established through genetic testing. The HSD17B3 gene is located on chromosome 9q22 and homozygous or compound heterozygous mutations in this gene cause 17b-HSD3 deficiency (8). As far as we know, there are 29 mutations identified in this gene at this time. These mutations include intronic splice sites, exonic deletions, missense and non-sense mutations (20,21). A great majority of these mutations have been identified in the Arab population living in the Gaza strip. The most common mutation identified in the Arab population is the p.Arg80Gln mutation, which is a point mutation in exon 3, codon 80 and 1The mutations previously identified in the Turkish population are c655-1;G-A, p.Ala188Val and c.777-783del_GATAACC mutations (3,22). Among these, c655-1;G-A is a splice junction mutation and disrupts splice acceptance site; p.Ala188Val is a missense mutation and inactivates the enzyme; and c.777-783del_GATAACC causes a 7 base pair deletion and frame shift and subsequently a truncated protein (23). The mutation identified in the current patient is a new mutation that has not been previously reported. In our case, a homozygous mutation c.761_762delAG corresponding to p.E254VfsX10 was identified in the HSD17B3 gene. A deletion of two nucleotides in exon 10 was found, which leads to a frame shift and subsequently to premature termination within the protein. Another important point of 17b-HSD3 deficiency is the lack of a phenotype-genotype correlation; different phenotypes have been reported in different individuals with the same genotype within the same family. Although the same homozygous mutation is seen in different individuals of the same pedigree, different T/Δ 4 ratios have been reported after hCG stimulation (18).

Sex assignment is a difficult and important decision in individuals with 17b-HSD3 deficiency, as is in other DSD cases. Transition to the male gender role is observed in a considerable amount (39-64%) of the individuals who have been raised as female, have not undergone gonadectomy and have experienced virilization in puberty (1,4,11,15,20,24,25). There are no reports of gender changes in cases raised as males (25). It has been observed that individuals who have been raised as females and have undergone gonadectomy during childhood are often satisfied with the female gender role and a very few of these individuals desire gender reassignment in the future (1,3,23). There is no association between the severity of the enzymatic defect and the adult social gender role and some cases are believed to have gender change possibly with the effect of social and cultural influences (1).

Some authors suggest that sex assignment and corrective surgeries at younger ages is more favorable for the child and the family to gain the gender role behavior, whereas some authors argue that it would be more favorable if the surgery is performed only after the child reaches an age to give his/her own consent and after obtaining his/her full consent (26,27). What is important here is that a delay is possible until the child reaches an age to disclose his/her choice and to assist the family and the doctors in making the right decision. The 2006 Chicago Consensus Meeting indicates that male gender assignment should be determined in individuals with 17b-HSD3 deficiency, but there is no spermatogenesis in individuals raised as male, even though early orchidopexy is performed and such individuals are infertile and have a risk of 28% for developing germ cell malignancy, so it should not be forgotten to closely monitor such individuals in this regard (28,29). Sex assignment should be determined in consideration of the social and cultural expectations of the society in which the family lives and religious convictions. If the male gender is assigned to the severely undervirilized individuals, such as the patient presented in this report, genital reconstruction may be difficult (30). There are also some opinions suggesting that the response of external genitalia to testosterone should be evaluated via testosterone injection before sex assignment and it would be appropriate to raise such individuals as male if there is an adequate response (30).

The important matter for sex assignment is that each case must be evaluated individually. In the current case, the patient’s parents indicated that they wanted to raise their child as female due to the completely female-appearing external genitalia and with the effect of the society’s sociocultural structure; therefore, an early gonadectomy was performed. During the follow-up of the patient, the necessary sex hormone replacements will be initiated in puberty and proper corrective surgeries such as cliteroplasty, vaginal dilatation, or vaginal constructive surgery can be performed, depending on the patient’s wishes.

In conclusion, 17b-HSD3 deficiency is an autosomal recessive form of 46,XY DSD. The diagnosis is made by appropriate endocrinological evaluation and a low T/Δ 4 ratio and confirmed by molecular genetic testing. 17b-HSD3 deficiency must be considered in all females presenting with inguinal hernia during infancy or childhood or having mild cliteromegaly and in all adolescent females presenting with virilization. Early and accurate diagnosis is important for the management and sex assignment of these patients, as well as for genetic counseling. Long-term follow-up is required for patients in terms of genitoplasty outcomes, sexual function, fertility and testicular malignancy risk.

## Figures and Tables

**Table 1:**
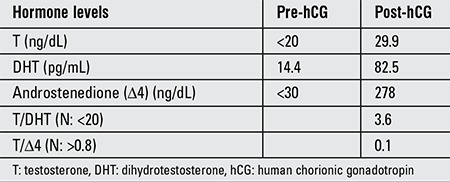
Serum androgen concentrations before and after human chorionic gonadotropin stimulation
